# Supplementary material for: Irp2 regulates insulin production through iron-mediated Cdkal1-catalyzed tRNA modification
Source: Nat Commun. 2020 Jan 15;11:296. doi: 10.1038/s41467-019-14004-5 (PMC6962211; doi:10.1038/s41467-019-14004-5)
Supplement: Supplementary file 1 — Supplementary Information [file 41467_2019_14004_MOESM1_ESM.pdf]

Supplementary Information

Irp2 regulates insulin production through iron-mediated Cdkal1-catalyzed tRNA modification

Ferreira et al.

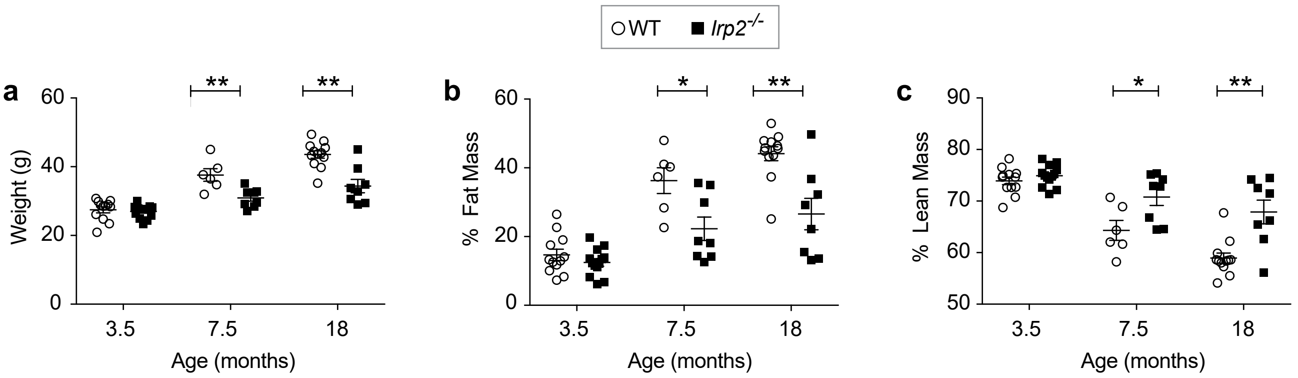

Supplementary Figure 1. Body composition of WT and *Irp2*$^{-/-}$ mice determined by nuclear magnetic resonance spectroscopy.

a-c Body weight (**a**), body fat mass (**b**) and body lean mass (**c**) of male WT and *Irp2*$^{-/-}$ mice at 3.5-, 7.5- and 18-months of age. Body composition analysis was measured using the Bruker Minispec NMR. Data are expressed as means ± s.e.m. and compared by unpaired two-tailed Student's *t*-test, *$p < 0.05$, **$p < 0.01$ relative to WT mice at each age.

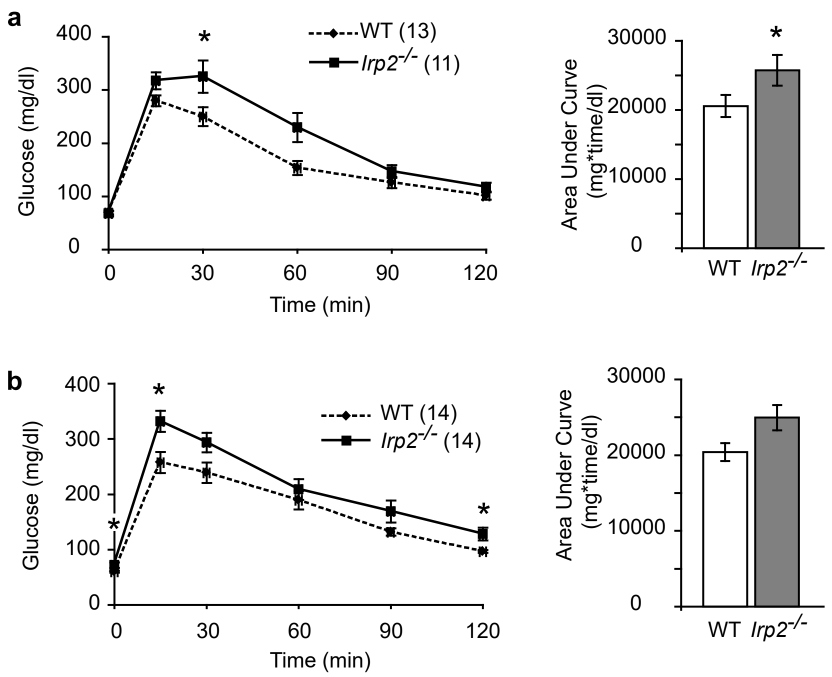

Supplementary Figure 2. Female *Irp2*$^{-/-}$ mice display glucose tolerance.
a-b, Intraperitoneal glucose tolerance tests (ipGTTs) (2 g glucose/kg body weight) were performed on female 10-week old (**a**) and 20-week old (**b**) WT and *Irp2*$^{-/-}$ mice. Glucose AUC for the data are shown. Data are the mean ± s.e.m., compared by unpaired two-tailed Students *t*-test. $p < 0.05$ relative to WT mice. Number of animals is shown in parentheses.

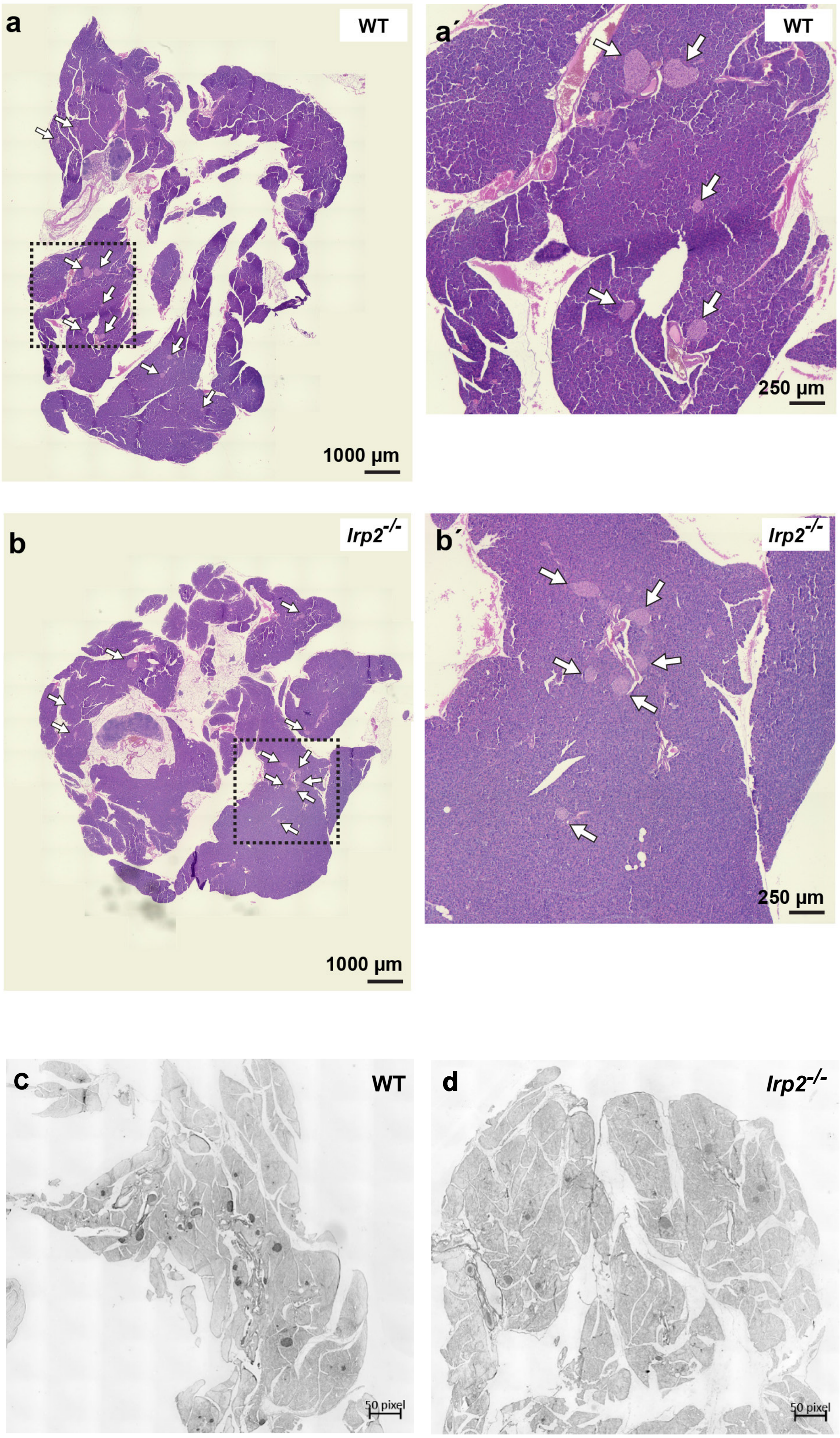

Supplementary Figure 3. H&E and insulin staining of paraffin-embedded pancreatic sections from 7.5-month old male WT and *Irp2*$^{-/-}$ mice.
a-b H&E staining and **c-d** insulin staining. Arrows indicate islets.

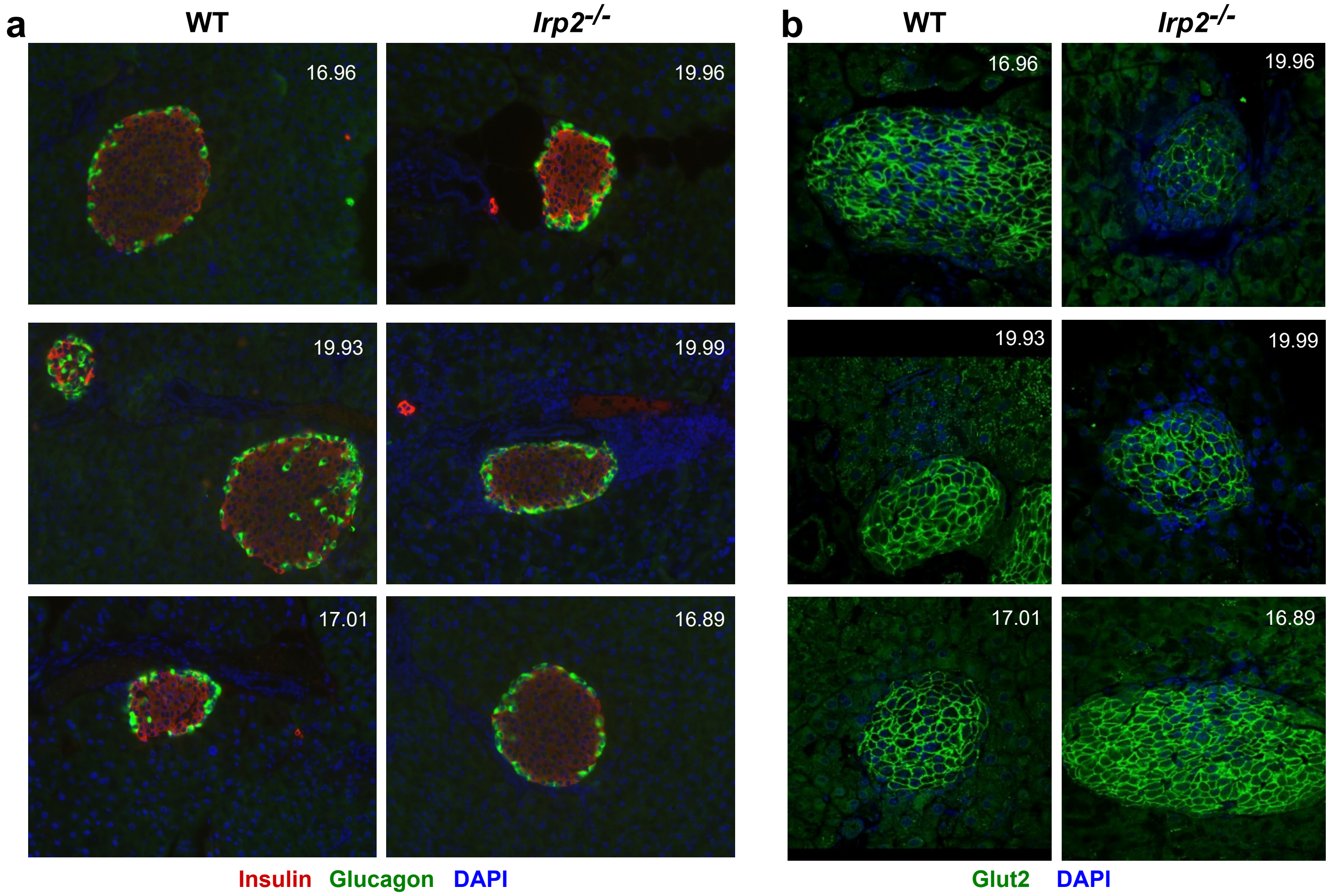

Supplementary Figure 4. **Glucagon, insulin and Glut2 staining show normal islet architecture and normal subcellular localization in *Irp2*$^{-/-}$ islets.**

a Glucagon and insulin costaining of WT and *Irp2*$^{-/-}$ islets revealed normal morphology with insulin-positive β cells uniformly distributed within the islet core and glucagon-positive α cells on the periphery. Representative paraffin-embedded pancreatic sections from 7.5-month old WT and *Irp2*$^{-/-}$ mice (n = 3 mice/genotype) were stained with insulin (red) and glucagon (green) antibodies. Nuclear DNA was detected by DAPI stain (blue). **b** Glut2 expression and subcellular localization in WT and *Irp2*$^{-/-}$ islets. Representative paraffin-embedded pancreatic sections from 7.5-month old WT and *Irp2*$^{-/-}$ mice (n = 3 mice/genotype) were stained with insulin (red) and Glut2 (green) antibodies. No change in Glut2 expression levels or subcellular localization was observed in *Irp2*$^{-/-}$ islets. Numbers in upper right of the panels represent animal number.

a

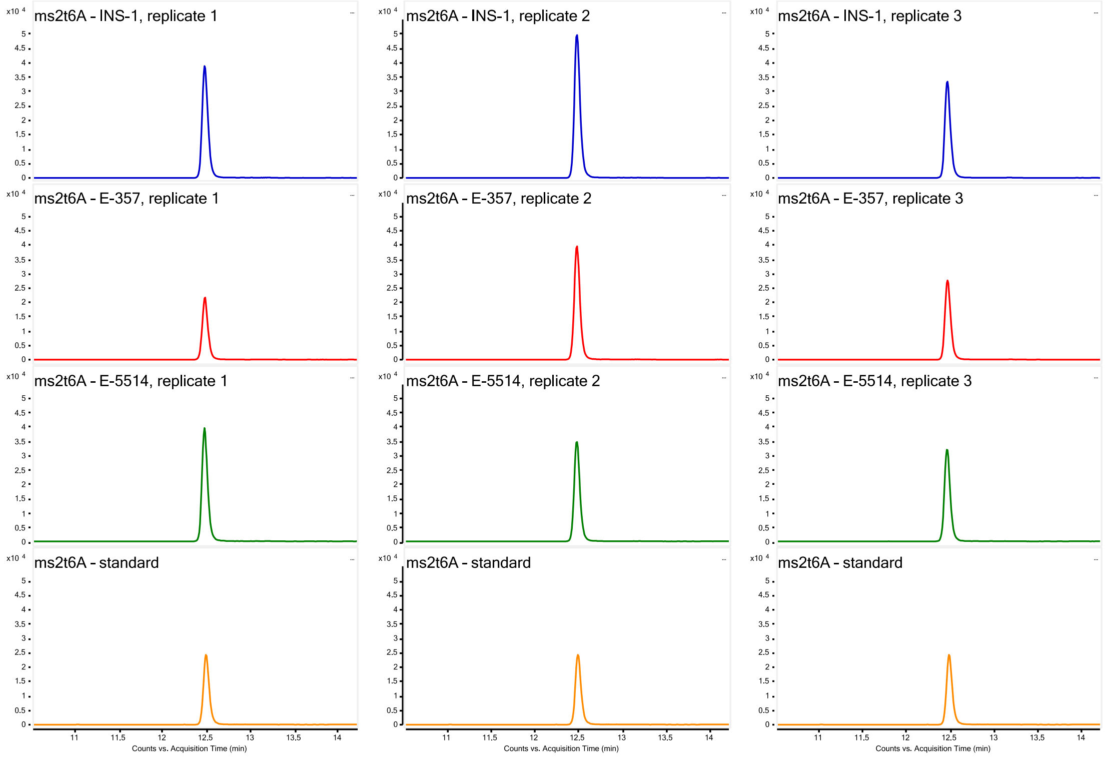

b

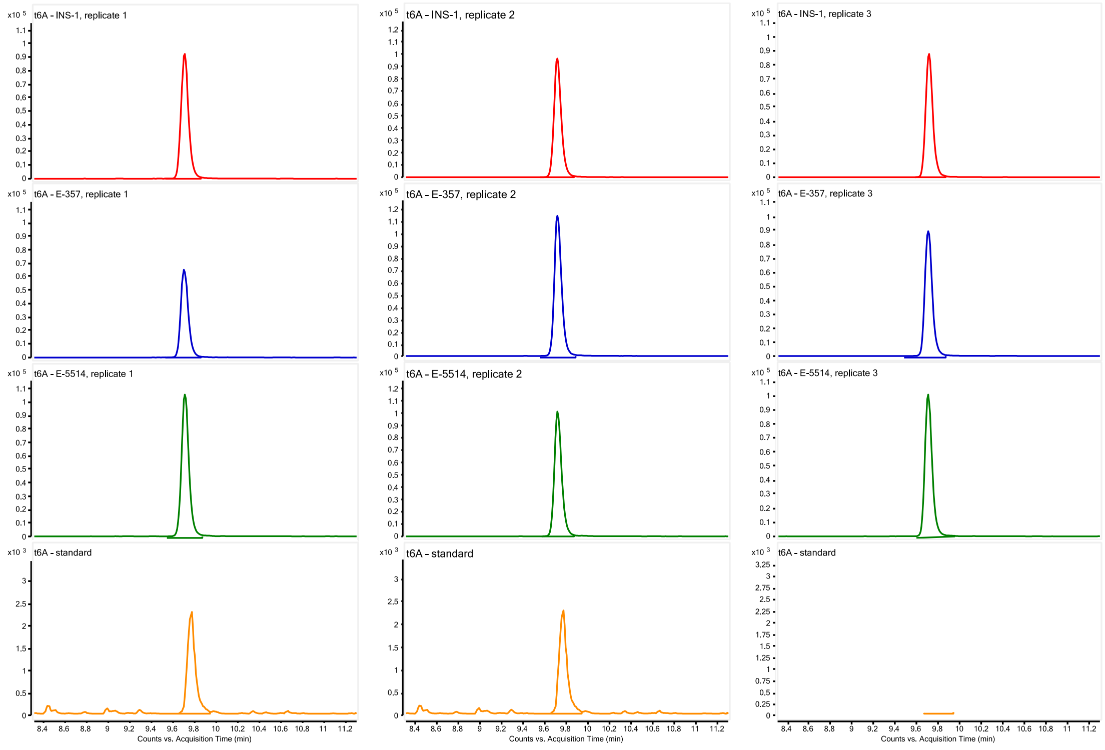

Supplementary Figure 5. Extracted ion chromatograms (EIC) showing ms^{2}2t^{2}A and t^{2}A signals in parental control and sgIrp2.1 and sgIrp2.2 INS-1 832/13 cell lines.
a, **b** EICs were obtained from chromatography-coupled tandem quadrupole mass spectrometric analysis of the m/z transitions 459→327 for ms^{2}t^{6}a (**a**) and 413→281 for t^{6}A (**b**). For panels **a** and **b**, the rows represent EICs for INS-1 (row 1), sgIrp2.1 (row 2; noted as "E-357" in the t^{6}A EIC) and sgIrp2.2 (row 3; noted as "E-5514" in the t^{6}A EIC) and for the synthetic standard (row 4). The columns represent EICS for three independent experiments analyzed at the same time. Peak areas derived from the EICs do not directly correlate with ms^{2}t^{6}A and t^{6}A levels since they have not been corrected to account for input RNA (based on areas of the canonical ribonucleosides; see Methods).

Supplementary Table 1. Plasma clinical chemistry parameters in male and female 3-month old WT and *Irp2*$^{-/-}$ mice

Group	WT male (10)	*Irp2*$^{-/-}$ male (10)	WT female (10)	*Irp2*$^{-/-}$ female (10)
Cholesterol [mg/dl]	125 ± 7.5	120.1 ± 7.45	93.1 ± 4.59	101.3 ± 6.18
Triglycerides [mg/dl]	126 ± 14.1	149.0 ± 7.0	85 ± 6.0	101.0 ± 9.0
NEFA [mmol/l]	0.89 ± 0.06	0.95 ± 0.06	0.67 ± 0.04	0.81 ± 0.06
LDL-Cholesterol [mg/dl]	13.72 ± 0.62	13.11 ± 0.75	13.79 ± 0.61	13.57 ± 0.66
HDL-Cholesterol [mg/dl]	80.46 ± 4.63	76.14 ± 4.71	57.8 ± 3.03	64.72 ± 3.76
Glucose [mg/dl]	187.7 ± 7.6	206.7 ± 8.1	167.7 ± 6.6	202.5 ± 10.7*
Ferritin [ng/ml]	25.7 ± 2.16	145.4 ± 2.66***	34.7 ± 11.55	135.6 ± 12.45***
Transferrin [mg/dl]	120.2 ± 1.23	117.0 ± 1.61	119.2 ± 2.04	122.2 ± 2.27
Iron [µg/dl]	104.5 ± 4.7	119.6 ± 5.99	133.8 ± 10.33	162.4 ± 11.27*

Clinical chemistry parameters were analyzed in plasma from random-fed, age-matched 3-month old male and female WT and *Irp2*$^{-/-}$ mice. Nonesterified fatty acids, NEFA. Data are expressed as means ± s.e.m.,compared by ANOVA, *$p < 0.05$; ***$p < 0.001$ relative to WT mice. Number of animals is shown in parentheses.

Supplementary Table 2. Hematological parameters in male and female 3-month old WT and *Irp2*$^{-/-}$ mice

Group	WT male (10)	*Irp2*$^{-/-}$ male (10)	WT female (10)	*Irp2*$^{-/-}$ female (10)
WBC [10^3/µl]	6.9 ± 0.32	7.5 ± 0.44	5.3 ± 0.56	5.7 ± 0.44
RBC [10^6/µl]	10.4 ± 0.09	10.1 ± 0.15	9.7 ± 0.11	9.4 ± 0.14
PLT [10^3/µl]	792 ± 19.2	816 ± 23.3	724 ± 17.1	700 ± 40.1
Hemoglobin [g/dl]	15.2 ± 0.13	13.3 ± 0.17***	14.6 ± 0.26	13.1 ± 0.22***
Hematocrit [%]	47.9 ± 0.33	42.3 ± 0.62***	45.1 ± 0.79	40.7 ± 0.68***
MCV [fl]	46 ± 0.21	42 ± 0.21***	46.5 ± 0.43	43.2 ± 0.29***
MCH [pg]	14.6 ± 0.09	13.2 ± 0.08***	15 ± 0.18	13.9 ± 0.12***
MCHC [g/dl]	31.7 ± 0.09	31.5 ± 0.1	32.3 ± 0.17	32.2 ± 0.13
RDW [% of MCV]	14.2 ± 0.05	14.5 ± 0.07**	14.3 ± 0.11	14.7 ± 0.1*
MPV [fl]	5.08 ± 0.08	5.05 ± 0.09	5.0 ± 0.08	5.19 ± 0.11

Hematological parameters were analyzed in random-fed, age-matched 3-month old male and female WT and *Irp2*$^{-/-}$ mice. Data are expressed as means ± s.e.m. and compared by ANOVA, *$p < 0.05$; **$p < 0.01$; ***$p < 0.001$ relative to WT mice. Number of animals is shown in parenthesis. Mean corpuscular volume (MCV), mean platelet volume (MPV), red blood cell distribution width (RDW), mean corpuscular hemoglobin (MCH), mean corpuscular hemoglobin concentrations (MCHC), red blood cell count (RBC), white blood cell count (WBC), platelet count (PLT).
